# Epidemiology of acute kidney injury and chronic kidney disease in the intensive care unit

**DOI:** 10.5935/0103-507X.20170061

**Published:** 2017

**Authors:** Darwin Tejera, Fernanda Varela, Daniela Acosta, Stephanie Figueroa, Sebastián Benencio, Cristina Verdaguer, Mauricio Bertullo, Federico Verga, Mario Cancela

**Affiliations:** 1 Associación Española, Montevideo, Uruguay.

**Keywords:** Acute kidney injury/epidemiology, Critcal care, Renal insufficiency, chronic, Mortality, Lesión renal aguda/epidemiología, Cuidados críticos, Insuficiencia renal crónica, Mortalidad

## Abstract

**Objective:**

To describe the epidemiology of acute kidney injury, its relationship to chronic kidney disease, and the factors associated with its incidence.

**Methods:**

A cohort study and follow-up were conducted in an intensive care unit in Montevideo, Uruguay. We included patients admitted between November 2014 and October 2015 who were older than 15 years of age and who had at least two measurements of serum creatinine. We excluded patients who were hospitalized for less than 48 hours, patients who died at the time of hospitalization, and patients with chronic renal disease who were on hemodialysis or peritoneal dialysis. There were no interventions. Acute kidney injury was defined according to the criteria set forth in Acute Kidney Injury Disease: Improving Global Outcomes, and chronic kidney disease was defined according to the Chronic Kidney Disease Work Group.

**Results:**

We included 401 patients, 56.6% male, median age of 68 years (interquartile range (IQR) 51-79 years). The diagnosis at admission was severe sepsis 36.3%, neurocritical 16.3%, polytrauma 15.2%, and other 32.2%. The incidence of acute kidney injury was 50.1%, and 14.1% of the patients suffered from chronic kidney disease. The incidence of acute septic kidney injury was 75.3%. Mortality in patients with or without acute kidney injury was 41.8% and 14%, respectively (p < 0.001). In the multivariate analysis, the most significant variables for acute kidney injury were chronic kidney disease (odds ratio (OR) 5.39, 95%CI 2.04 - 14.29, p = 0.001), shock (OR 3.94, 95%CI 1.72 - 9.07, p = 0.001), and severe sepsis (OR 7.79, 95%CI 2.02 - 29.97, p = 0.003).

**Conclusion:**

The incidence of acute kidney injury is high mainly in septic patients. Chronic kidney disease was independently associated with the development of acute kidney injury.

## Abstract (es)

**Objetivo:**

Describir la epidemiología de la injuria renal aguda, la relación con la enfermedad renal crónica y los factores asociados a su incidencia.

**Métodos:**

Estudio de cohorte y seguimiento en una unidad de terapia intensiva de Montevideo - Uruguay. Se incluyeron pacientes ingresados entre noviembre 2014 a octubre 2015, mayores de 15 años con dos mediciones de creatinina sérica. Se excluyeron pacientes con menos de 48 horas de internación o fallecidos en ese tiempo y portadores de enfermedad renal crónica en hemodiálisis o diálisis peritoneal. No hubo intervenciones. La injuria renal aguda se definió según criterios *Acute Kidney Injury Disease Improving Global Outcomes* y la enfermedad renal crónica según *Cronic Kidney Disease Work Group*.

**Resultados:**

Se incluyeron 401 pacientes, sexo masculino 56,6%, mediana de edad 68 (rango intercuartílico - RIC 51 - 79) años. El diagnóstico al ingreso fue sepsis grave 36,3%, neurocrítico 16,3%, politrauma 15,2% y otros 32,2%. La incidencia de injuria renal aguda fue de 50,1%. El 14,1% eran portadores de enfermedad renal crónica. La incidencia de injuria renal aguda séptica fue de 75,3%. La mortalidad en los pacientes con o sin injuria renal aguda fue de 41,8 y 14% respectivamente (p < 0,001). En el análisis multivariado las variables de mayor significación para la injuria renal aguda fueron enfermedad renal crónica (*odds ratio* - OR 5,39 IC95% 2,04 - 14,29 p = 0,001), shock (OR 3,94 IC95% 1,72 - 9,07 p = 0,001) y sepsis grave (OR 7,79 IC 95% 2,02 - 29,97 p = 0,003).

**Conclusión:**

La incidencia de injuria renal aguda es elevada principalmente en pacientes sépticos. La enfermedad renal crónica se asoció de forma independiente al desarrollo de injuria renal aguda.

## INTRODUCTION

Acute kidney injury (AKI) is a serious complication in critically ill patients and is associated with increased morbidity and mortality, increased hospitalization time and care cost, and long-term development of chronic kidney disease (CKD).^(1,2)^ The overall incidence of AKI in the critical patient population varies according to the definition used and the population studied and ranges from 20% to 50%.^(1,2)^

In recent years, studies in different regions have found that CKD is a strong risk factor for the development of AKI, mainly in septic patients. Currently, CKD is found in 30% of patients who develop AKI in the intensive care unit (ICU).^(1,3)^

The current diagnosis of AKI is based on the determination of changes in serum creatinine (CrS) and urine output.^(4)^ In the last decade, both the diagnosis and the classification of AKI have been standardized according to the criteria set forth in Risk, Injury, Failure, Loss, End-Stage Kidney Disease (RIFLE), Acute Kidney Injury Network (AKIN), and Acute Kidney Injury Kidney Disease: Improving Global Outcomes (AKI-KDIGO).^(3,5)^

AKI is an independent risk factor associated with mortality in critically ill patients, mainly as a component of multiple organ dysfunction in patients with severe sepsis.^(1,3,6)^

The objectives of this study are to evaluate the evolution and mortality of various subgroups of patients with AKI, to analyze the clinical characteristics of septic and non-septic AKI, to determine the incidence of chronic renal failure (CRF) and its relationship to AKI, and to assess factors associated with higher mortality.

## METHODS

A prospective, follow-up cohort study was performed at an ICU in the city of Montevideo, Uruguay. We included all patients who entered the ICU during the period from November 2014 to October 2015 who were older than 15 years of age and who had at least two measurements of serum creatinine (CrS). Patients who remained in the ICU for less than 48 hours or who died at that time and patients with CKD who were on hemodialysis or peritoneal dialysis were excluded.

The study was presented to and accepted by the ethics committee of the institution (*Asociación Española*). Because it was an anonymous study without interventions, the committee did not request informed consent for performing the study. The manuscript was prepared according to the STROBE (Strengthening the Reporting of Observational Studies in Epidemiology) guidelines for the communication of observational studies.^(7)^

In the collection of information, variables of interest were included in a pre-established database during ICU admission. These variables included demographic data (age and sex), personal medical history, use of nephrotoxic drugs in the month prior to admission and during ICU stay, CKD, Acute Physiology and Chronic Health Evaluation II (APACHE II) score on admission, use of vasopressors and mechanical ventilation, nephrological history (previous AKI, proteinuria, and hematuria), development of AKI in the ICU and need for renal replacement therapy (RRT), creatinine at baseline and on ICU admission, paraclinical examination on admission (blood count, urea, albuminemia, and blood gases), associated ionic alterations during progression, and mortality in the ICU and hospital.

The main variables were defined according to the diagnostic criteria currently set forth in international guidelines. The diagnosis and classification of AKI was performed according to the AKI-KDIGO criteria, as shown in table 1.^(5)^ The baseline creatinine considered was the last stable measurement in the patient's clinical history prior to admission to the ICU. CKD was defined according to CKD-KDIGO criteria and is indicated by the presence of a glomerular filtrate < 60 mL/min/1.73 m^2^ in the last three months associated with renal damage markers such as albuminuria, urinary sediment abnormalities, ionic alterations secondary to tubular disorders, abnormal histology, structural alterations in imaging studies, and/or a history of renal transplantation.^(8)^ Patients were not included in the study if the diagnosis of CKD was uncertain based on analysis of their previous medical histories.

**Table 1:** Definition and classification of acute kidney injury according to AKI-KDIGO criteria

**Definition**		
1) CrS increase > 0.3 mg/dL in 48 hours.		
2) CrS increase > 1.5 from baseline in the last 7 days.		
3) Diuresis < 0.5 mL/kg/h for 6 hours.		
**Classification**	**Serum creatinine**	**Diuresis**
Stage 1	Increased CrS > 0.3 mg/dL or CrS 1.5 - 1.9 baseline	< 0.5 mL/kg/h for 6 to 12 hours
Stage 2	CrS 2.0 - 2.9 baseline	< 0.5 mL/kg/h for > 12 hours
Stage 3	CrS 3 baseline level or need for renal replacement therapy	< 0.3 mL/kg/h for > 24 hours or anuria > 12 hours

CrS - serum creatinine.

### Statistical analysis

Statistical processing was performed using the Statistical Package for Social Sciences (SPSS) version 18. Descriptive statistical analysis was performed for the distribution of absolute and relative frequencies of the variables studied. Descriptive measures such as the mean and standard deviation (SD) were calculated for the quantitative variables; for the non-normal quantitative variables, the median and interquartile range (IQR) were calculated. For the analysis of association of categorical variables, the chi square test or Fisher's exact test was performed when appropriate; for comparisons of inter-group means, the t-test was applied for independent groups, and 95% confidence intervals (95%CI) were calculated for the mean. A value of p<0.05 was considered statistically significant. A risk analysis was performed for the incidence of AKI by calculating the odds ratio (OR) and its 95% CI. The criterion used for the multivariate analysis of AKI was to include the variables that were statistically significant in the bivariate analysis (p < 0.05). A binary logistic regression model was used.

## RESULTS

During the study period, 541 patients were admitted to the ICU; of these, 401 met the inclusion criteria. The median age of the included patients was 68 years (IQR 51 - 79 years) with a range of 18 to 92 years; 56.6% were male. The median APACHE II score at admission was 16 (IQR 11 - 22) and ranged from 2 to 49. The diagnosis at admission to the ICU was as follows: severe sepsis, 36.3%; neurocritical, 16.3%; polytrauma, 15.2%; and other causes, 32.2%. The main co-morbidities were arterial hypertension (50.1%), smoking (27.6%), heart disease (26.3%), neoplasia (20.3%; solid 83.8% and hematologic 16.2%), dyslipidemia (19.8%), and diabetes mellitus (18.6%). Two or more comorbidities were present in 61.7% of the patients. A total of 56 patients (14.1%) were carriers of CKD. Sixteen percent had a prior history of AKI, 12.2% had proteinuria and/or hematuria, and 12.5% ​​had other nephrological pathologies, mainly obstructive (lithiasic or prostatic).

The median number of days spent in the ICU was 3 (IQR 2 - 8), and the median number of days on mechanical ventilation in the population that required ICU stays was 3 (IQR 1 - 9). In patients with and without AKI, the mean ICU stays were 4 days (IQR 2 - 13) and 3 days (IQR 1 - 5), respectively (p < 0.001). The median days on mechanical ventilation in patients with and without AKI were 4 (IQR 2 - 13) and 2 (IQR 1 - 6), respectively (p < 0.001). The overall mortality of the ICU population was 28.1% (n = 112), and hospital mortality was 29.4% (n = 118).

**Figure 1:**
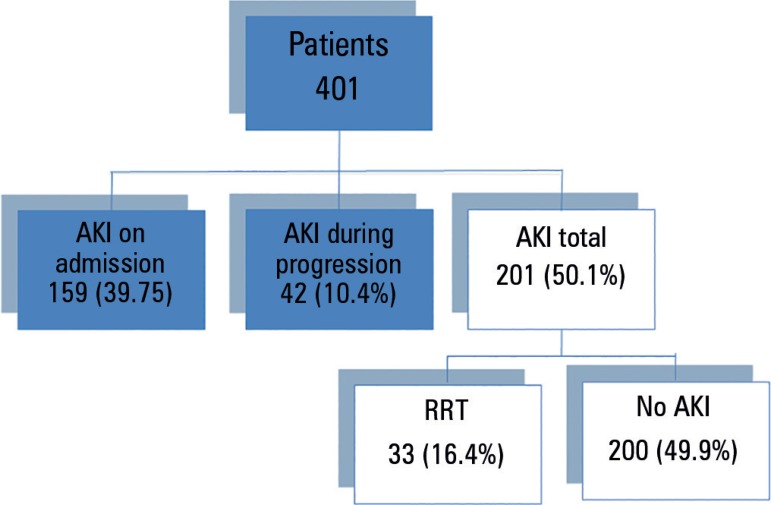
Incidence of acute kidney injury in the study population.

**Figure 2:**
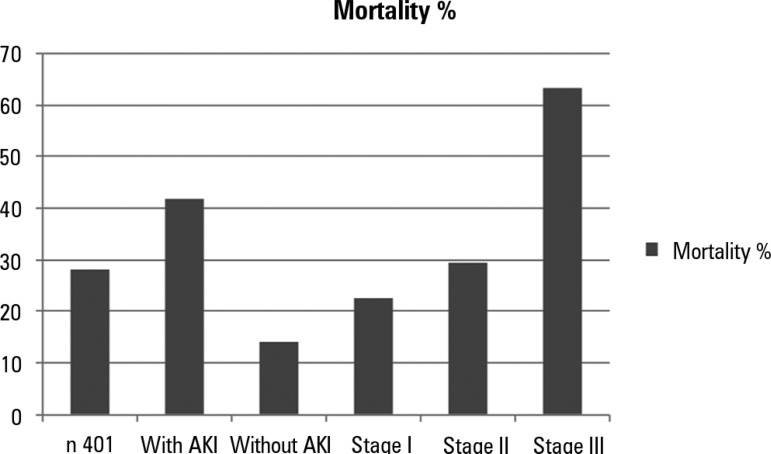
Relationship between mortality in the intensive care unit, incidence of acute kidney injury and stage according to AKI-KDIGO. AKI - acute kidney injury. p < 0.001.

The overall incidence of AKI in the population was 50.1%. Figure 1 shows the general incidence of AKI on admission and during progression in the ICU. The AKI classification according to AKI-KDIGO criteria was stage I in 37.8% of the patients, stage II in 17.4% of the patients and stage III in 44.8% of the patients. The relationship between mortality and the incidence and classification of AKI is presented in figure 2. Table 2 shows the general characteristics of patients with and without AKI.

**Table 2:** Clinical characteristics of the population of patients with and without acute kidney injury

Variable	All patients	Patients with AKI	Patients without AKI	p value
N (%)	401 (100)	201 (50.1)	200 (49.9)	
Age	68 (51 - 79)	72 (60 - 81)	64 (45 - 76)	< 0.001
Male	227 (56.6)	113 (56.2)	114 (57)	0.477
APACHE II	16 (11 - 22)	21 (14 - 27)	13 (9 - 17)	< 0.001
Arterial hypertension	201 (50.1)	121 (60.2)	79 (39.5)	< 0.001
Diabetes mellitus	74 (18.6)	48 (23.8)	26 (13)	0.004
Cardiopathy	104 (26.3)	68 (33.8)	36 (18)	< 0.001
Heart failure	56 (14.2)	41 (20.4)	15 (7.5)	< 0.001
COPD	30 (7.6)	19 (9.4)	11 (5.5)	0.091
Stroke	39 (9.8)	17 (8.4)	22 (11)	0.250
Obesity	45 (11.4)	31 (15.4)	14 (7)	0.006
Smoking	109 (27.6)	56 (27.8)	52 (26)	0.423
Peripheral vascular disease	16 (4.1)	10 (4.9)	6 (3)	0.222
Alcoholism	37 (9.4)	12 (5.9)	25 (12.5)	0.018
Dyslipidemia	78 (19.8)	35 (17.4)	43 (21.5)	0.195
Hyperuricemia	38 (9.7)	24 (11.9)	14 (7)	0.060
Hepatopathy	17 (4.3)	6 (2.9)	11 (5.5)	0.161
Neoplasia	80 (20.3)	38 (18.9)	42 (21)	0.363
Chronic kidney disease	56 (14.1)	48 (23.8)	8 (4)	< 0.001
Previous nephrological pathology	48 (12.2)	36 (17.9)	12 (6)	< 0.001
Previous AKI	63 (16)	47 (23.4)	16 (8)	< 0.001
Proteinuria and/or hematuria	48 (12.2)	35 (17.4)	13 (6.5)	0.001
Prior nephrotoxics	129 (32.4)	77 (38.3)	52 (26)	0.005
NSAIDs	34 (8.6)	19 (9.4)	15 (7.5)	0.296
ARA II	36 (9.1)	21 (10.4)	15 (7.5)	0.191
ACE inhibitors	48 (12.1)	28 (13.9)	20 (10)	0.141
Contrast media	33 (8.3)	22 (10.9)	11 (5.5)	0.035
Diuretics	33 (8.3)	23 (11.4)	10 (5)	0.013
Aminoglucosides	14 (3.5)	10 (4.9)	4 (2)	0.086
Vancomycin	7 (1.8)	6 (2.9)	1 (0.5)	0.061
Colistin	9 (2.3)	7 (3.4)	2 (1)	0.087
Diagnosis on admission				
Sepsis	146 (36.4)	110 (54.7)	36 (18)	< 0.001
Neurocritical	65 (16.2)	15 (7.4)	50 (25)	< 0.001
Polytrauma	61 (15.2)	18 (8.9)	43 (21.5)	< 0.001
Other	129 (32.2)	58 (28.8)	71 (35.5)	< 0.001
CrS baseline (mg/dL)	0.88 (0.72 - 1.06)	1.0 (0.81 - 1.20)	0.8 (0.67 - 0.92)	< 0.001
CrS admission UTI (mg/dL)	0.98 (0.73 - 1.62)	1.6 (1.10 - 2.80)	0.78 (0.62 - 0.93)	< 0.001
Urea (mg/dL)	46 (30 - 79)	77 (46 - 114)	32 (25 - 45)	< 0.001
Diuresis (mL)	1300 (800 - 1800)	950 (400 - 1500)	1500 (1200 - 1800)	< 0.001
Na^+^ (meq/L)	138 (134 - 141)	138 (133 - 142)	137 (134 - 140)	0.513
K^+^ (meq/L)	3.9 (3.6 - 4.4)	4.1 (3.7 - 4.6)	3.8 (3.5 - 4.1)	< 0.001
Ca^+^^+^ (mmoL/L)	1.10 (1.06 - 1.17)	1.10 (1.03 - 1.15)	1.12 (1.08 - 1.17)	0.001
Cl^-^ (meq/L)	105 (101 - 110)	106 (101 - 111)	104 (100 - 109)	0.012
Glycemia (g/dL)	1.50 (1.19 - 1.87)	1.54 (1.20 - 2.03)	1.50 (1.16 - 1.79)	0.088
Hemoglobin (g/dL)	11.0 (9.4 - 12.6)	10.6 (9.0 - 12.1)	11.3 (9.9 - 12.9)	0.001
Platelets (x10^9^/L)	195 (134 - 259)	194 (125 - 264)	198 (145 - 254)	0.438
Leukocytes (x10^9^/L)	12.0 (8.7 - 16.1)	12.6 (8.8 - 18.1)	11.5 (8.4 - 15.4)	0.062
Albuminemia (g/dL)	3.0 (2.6 - 3.5)	2.9 (2.5 - 3.4)	3.0 (2.8 - 3.6)	< 0.001
pH	7.35 (7.29 - 7.40)	7.32 (7.25 - 7.39)	7.28 (7.33 - 7.41	< 0.001
PaO_2_ (mmHg)	145 (98 - 215)	129 (91 - 205)	161 (109 - 222)	0.012
PaCO_2_ (mmHg)	38 (33 - 44)	37 (32 - 46)	39 (34 - 43)	0.318
HCO_3_^-^ (meq/L)	21 (18 - 23)	19 (16 - 22)	22 (20 - 24)	< 0.001
Lactate (meq/L)	2.0 (1.3 - 3.1)	2.3 (1.6 - 4.9)	1.7 (1.1 - 2.4)	< 0.001
Mechanical ventilatory assistance	290 (72.9)	163 (81.1)	127 (63.5)	< 0.001
ARDS	107 (26.9)	82 (40.8)	25 (12.5)	< 0.001
Vasopressors/inotropics	154 (38.6)	120 (59.7)	29 (14.5)	< 0.001
Diuretics day 1	57 (14.4)	40 (19.1)	17 (8.5)	0.001
Nephrotoxics in ICU	147 (36.8)	77 (38.3)	70 (35)	0.267
ACE inhibitors	33 (8.3)	20 (9.9)	13 (6.5)	0.134
ARA II	4 (1)	2 (1)	2 (1)	0.688
NSAIDs	77 (19.3)	29 (14.4)	48 (24)	0.011
Contrast medium	17 (4.3)	11 (5.4)	6 (3)	0.161
Vancomycin	23 (5.8)	20 (9.9)	3 (1.5)	< 0.001
Aminoglucosides	29 (7.3)	18 (8.9)	11 (5.5)	0.123
Colistin	23 (5.8)	18 (8.9)	5 (2.5)	0.004
Transfusion	129 (32.6)	93 (46.2)	36 (18)	< 0.001
Diuretics in evolution	112 (28.3)	79 (39.3)	33 (16.5)	< 0.001
ICU mortality	112 (28.1)	84 (41.8)	28 (14)	< 0.001
Hospital mortality	118 (29.4)	85 (42.2)	33 (16.5)	< 0.001

AKI - acute kidney injury; APACHE II - Acute Physiology and Chronic Health Evaluation II; COPD - chronic obstructive pulmonary disease; NSAIDs - non-steroidal anti-inflammatory drugs; ARA II - angiotensin II receptor antagonists; ACE inhibitors - inhibitors of angiotensin-converting enzyme; CrS - serum creatinine; ICU - intensive care unit; Na^+^ - sodium; K^+^ - potassium; Ca^+^^+^ - calcium; Cl^-^ chloride; pH - concentration of hydrogen ions; PaO_2_ - partial arterial oxygen concentration; PaCO_2_ - partial pressure of carbon dioxide; HcO_3_ - bicarbonates; ARDS - acute respiratory distress syndrome. Values are expressed as N (%) and the median (interquartile range).

In patients diagnosed with severe sepsis, the incidence of AKI was 75.3%. Table 3 compares the characteristics of the patient populations with septic and non-septic AKI.

**Table 3:** Comparison of patients with acute septic and non-septic kidney injury

Variable	Septic AKI	Non-septic AKI	p value
N	110	91	
Age	74 (61 - 82)	70 (57 - 80)	0.292
Male	56 (50.9)	57 (62.6)	0.063
APACHE II	22 (17 - 29)	18 (13 - 24)	0.001
CrS baseline (mg/dL)	1 (0.81 - 1.29)	1 (0.81 - 1.14)	0.883
CrS admission UTI (mg/dL)	1.78 (1.15 - 3.27)	1.52 (1.05 - 2.18)	0.079
Urea (mg/dL)	0.82 (0.56 - 1.22)	0.72 (0.43 - 1.07)	0.56
Diuresis (mL)	800 (300 - 1400)	1100 (500 - 1800)	0.007
Stage I	31 (28.2)	45 (49.5)	0.006
Stage II	20 (18.2)	15 (16.5)	0.006
Stage III	59 (53.6)	31 (34.1)	0.002
RRT	20 (18.1)	13 (14.2)	0.310
Mechanical ventilation	93 (85.3)	70 (76.9)	0.090
Vasopressors	76 (69.1)	40 (44.0)	< 0.001
ICU mortality	54 (49.1)	30 (32.9)	0.015

AKI - acute kidney injury; APACHE II - Acute Physiology and Chronic Health Evaluation II; CrS - serum creatinine; ICU - intensive care unit; RRT - renal replacement therapy. Values are expressed as the median (interquartile range and N (%).

In the group of patients with AKI, 33 patients required RRT (16.4%). Mortality in the groups with and without RRT was 54.5% and 41.0%, respectively (p = 0.108). Table 4 shows the main hydroelectrolytic alterations in patients with AKI. Table 5 shows the results of multivariate analysis of the incidence of AKI.

**Table 4:** Hydroelectrolytic alterations in patients with and without acute kidney injury

	All N (%)	AKI N (%)	No AKI N (%)	p value
Hyponatremia	186 (46.9)	99 (53.2)	87 (46.8)	0.12
Hypernatremia	99 (24.9)	71 (71.7)	28 (28.3)	< 0.001
Hyperchloremia	283 (71.1)	161 (56.9)	122 (43.1)	< 0.001
Hypocalcemia	234 (59.1)	133 (56.8)	101 (43.2)	0.001

AKI - acute kidney injury.

**Table 5:** Multivariate analysis of factors associated with the incidence of acute kidney injury

Variable	OR	95%CI	p value
Arterial hypertension	1.96	1.03 - 3.74	0.041
Chronic kidney disease	5.39	2.04 - 14.29	0.001
Hypernatremia	2.09	1.02 - 4.31	0.045
Transfusions	2.89	1.47 - 5.68	0.002
Shock	3.94	1.72 - 9.07	0.001
ARDS	2.52	1.24 - 5.12	0.010
APACHE II ≥ 17	1.06	1.01 - 1.12	0.010
Sepsis	7.79	2.02 - 29.97	0.003

OR - odds ratio; CI - confidence interval; ARDS - acute respiratory distress syndrome; APACHE II - Acute Physiology and Chronic Health Evaluation II.

## DISCUSSION

Acute kidney injury is a clinical syndrome associated with multiple diseases and pathophysiological mechanisms, including hypoxia, ischemia-reperfusion, and inflammation, among others.^(1)^ Contemporary studies using definitions based on urea and diuresis have generally found minor incidences of AKI. The study by Uchino et al.,^(9)^ which was conducted in 2000-2001 and was based on these parameters, reported an incidence of AKI in the ICU of 5.7%.

In our study, the overall incidence of AKI in the ICU was 50.1%. This incidence is similar to that reported in recent studies in which the AKI-KDIGO criteria were also used to define AKI. However, publications on the epidemiology of AKI report highly variable incidences ranging from 26% to 67%.^(1,3,5,9,10)^ The reported incidence depends both on the population studied and on the diagnostic criteria used (RIFLE, AKIN, or AKI-KDIGO). A European study of more than 50,000 patients found an incidence of 9% for hospital AKI using the AKI-KDIGO criteria.^(11)^ The Italian multicenter study by Piccinni et al., which used the RIFLE criteria, found an incidence of 65.8% for AKI.^(12)^ The work of Zhou et al.,^(13)^ in which AKI was defined according to the AKIN criteria, found an incidence of 34.1%. The recent multinational study published by Hoste et al.,^(14)^ in which the AKI-KDIGO criteria were used, reports that there are significant differences in the incidence and etiology of AKI. The overall incidence reported was 57.3%. In the same study, the incidence of AKI in a subgroup of 244 patients in South America was 53.2%, similar to our results. A recent study conducted in Brazil reported no difference in the prediction of mortality when RIFLE, AKIN, or KDIGO criteria were used to establish the diagnosis of AKI.^(15)^ Using the AKI-KDIGO definition, Srisawat et al. found an incidence of 32% for AKI using CrS as a criterion, with a hospital mortality of 27%.^(16)^

With respect to the classification of AKI, the largest group of patients was classified as stage III (44.8%); stage I and stage II were of lower incidence. This finding can be partially explained by the rapid clinical deterioration of patients who suffer an AKI. In addition, most patients entered the ICU with a diagnosis of AKI (39.7%), and a minor subgroup developed it in the ICU, suggesting that clinical deterioration may be more significant and may proceed more rapidly prior to admission.

Table 2 shows that certain variables, including advanced age, APACHE II, and the presence of comorbidities, were significantly associated with an increased incidence of AKI. An association between comorbidities and the incidence of AKI was mainly detected for cardiovascular risk factors (arterial hypertension and diabetes mellitus), the presence of heart disease and heart failure, alcoholism, and obesity. The results also show a clear association of the development of AKI with a history of nephrourological pathologies such as CKD, lithiasic and/or tumoral pathology, previous AKI, proteinuria, and/or hematuria and with the use of nephrotoxic drugs, mainly the use of contrast medium and diuretics, both previous to admission and in the ICU. In addition, this association can be explained by a lower functional reserve and an increased risk of AKI in this subgroup of patients. Risk factors such as age, hypovolemia, diabetes mellitus, chronic heart, and respiratory disease are considered to predispose individuals to the development of AKI.^(17)^

In the present study, we found a significant association between the development of AKI and mortality (Table 2). In addition, mortality was found to be directly related to the classification of AKI, as shown in figure 2. Previous studies of the epidemiology of AKI showed that the risk of death increased significantly with the level of CrS^(10)^ and with the stage of progression.^(18)^ The FRAMI study, which was conducted in 43 Spanish ICUs, showed that the occurrence of AKI in critically ill patients is independently associated with higher mortality, with an OR of 2.5.^(6)^ Wide variability in outcomes regarding incidence and mortality is also influenced by the patients' prior functional status and by the presence or absence of pathological conditions associated with AKI.^(19)^

Although the population studied in the present work is heterogeneous, two groups of patients with AKI, septic patients and non-septic patients, can be distinguished, as shown in table 3. The incidence of AKI was significantly higher in septic patients than in non-septic patients.

It is known that the development of AKI during sepsis is an independent risk factor associated with higher mortality in critically ill patients.^(9)^ Sepsis and septic shock are the cause of approximately 50% of cases of AKI in critically ill patients^(9)^, and major surgery ranks second as a cause in terms of frequency.^(19)^ A study conducted in Africa also reported that the most common etiology of AKI was sepsis in 54.9% of cases.^(20)^ Other work in China reported an incidence of AKI of 54.7% in critically ill patients, with sepsis and septic shock being the most frequent etiology at 49.2%.^(21)^

In septic AKI, associated mortality rates of 50-60% have been reported.^(22)^ In our study, we found a crude mortality rate of 49.1% in septic AKI. In the NEFROINT study, septic AKI had an incidence of 77.8% and a mortality rate of 38%.^(12)^ In septic patients, the results and evolution of AKI are also influenced by the therapeutic measures implemented, such as the initial replacement and quality of fluids administered, the use of vasopressor drugs, the overload, and accumulated fluid balance, among others.^(23)^ In addition, we found that mortality in septic AKI was significantly higher than that in non-septic AKI. In a recent study comparing the clinical features of septic AKI versus non-septic AKI, similar outcomes were reported, with a significantly higher mortality in septic than in non-septic AKI.^(24)^ In patients with sepsis, multiple pathogenic factors such as renal hypoperfusion, altered intrarenal hemodynamics, oxidative stress, inflammation, mitochondrial dysfunction, and others are involved in the development and progression of AKI.^(25)^ The prognosis of septic AKI also depends on the degree of recovery of renal function. In the work of Herrera-Gutierrez et al.,^(6)^ the total or partial reversibility of AKI in the first hours of ICU admission was associated with decreased mortality in patients with septic shock.

Ionic alterations such as hyperchloremia, hypocalcemia, and hypernatremia were also associated with an increase in the incidence of AKI, as seen in table 4. Hydroelectrolytic alterations and their magnitudes are also manifestations of the severity of the critical illness and the treatment measures, mainly the administration of fluids.

On the other hand, in the presented work, we found that there is a marked relationship between the incidence of AKI and the presence of CKD.

In patients with AKI, a high percentage (16.4%) required implementation of RRT. Mortality was somewhat higher in the group requiring RRT, but the difference was not statistically significant. Mortality was associated with the incidence and stage of AKI but not with the requirement for RRT. The observed high incidence of patients with AKI who required RRT can be explained in part by the fact that the study population includes many older individuals and by the severity of the critical illness of the patients in the study; in addition, a large group of the patients in the study were carriers of CKD (14.1%) and therefore had already suffered deterioration in renal function and glomerular filtration and had less functional reserve.

On the other hand, a higher incidence of AKI has been observed during the last decade, and more liberal criteria for the initiation of RRT are applied than were used previously.^(14,26)^ Sepsis and other critical conditions have been shown to be important risk factors for the development of AKI requiring RRT.^(27)^ One study in the United States mentions that despite an increase in the incidence of AKI requiring RRT, hospital mortality in this group of patients has decreased.^(28)^

Table 5 shows that arterial hypertension, CRF, hypernatremia, transfusions, shock, acute respiratory distress syndrome (ARDS), elevated APACHE II, and sepsis were risk factors associated with a higher incidence of AKI. It is noteworthy that CKD was the variable with the highest percentage of association, which makes it a strong predictor for the development of AKI in critically ill patients. This should be taken into account when administering nephrotoxic drugs and using contrast media. Current strategies for the prevention and management of AKI, including the type of fluid used for resuscitation and the timing of RRT onset, have been described, mainly with reference to septic patients with multiorgan dysfunction and CKD.^(29)^ In addition, risk factors for perioperative AKI similar to those found in our study have been identified.^(30)^

The main relevance and strength of this study is its prospective design and the inclusion of patients in whom CrS was measured in the three months prior to ICU admission, allowing a more accurate diagnosis of the previous renal status of the study participants. The use of baseline CrS leads to an increase in the occurrence of AKI compared to studies in which it is calculated retrospectively using the MDRD formula. An adequate number of patients were recruited, and CrS and diuresis were both considered in the diagnosis of AKI.

Among the limitations of the study are, first, that it was performed in a single center; second, the demographic characteristics show that it involved a population of elderly patients. This means the results cannot be generalized to all age groups. In addition, we know that the incidence of AKI, as well as its etiology, is influenced by geographical and socioeconomic factors. Another limitation of this study is that long-term data on the progression of AKI, the need for RRT and mortality were not collected. However, the results are comparable to those reported in a number of publications in which the reported incidence of AKI and the associated mortality are highly variable.

Future perspectives point to the development of specific methods for the assessment and treatment of critically ill patients. These methods include adequate monitoring of CKD and diuresis, daily assessment of nephrotoxic drug indications and use, use of balanced crystalloids, and control of potassium supplements with the aim of reducing the incidence of AKI, allowing early diagnosis and improving the outcomes of patients with AKI in the ICU.^(31)^ New areas of interest in research that address the prevention and early diagnosis of AKI through nephroprotective drugs and the use of biomarkers of renal damage are currently being pursued.^(32)^ The implementation of these measures requires the development of new studies that evaluate the results.

## CONCLUSION

The incidence of acute kidney injury in critical patients is high primarily in septic patients. Both the presence of acute kidney injury and its stage are significantly associated with higher mortality. The prevalence of chronic kidney disease in critically ill patients is high. Both chronic kidney disease and a history of other renal pathologies are risk factors for the development of acute kidney injury. The requirement for renal replacement therapy was not significantly associated with increased mortality in patients with acute kidney injury. The results of this study are similar to those of other studies in our region that have used the same diagnostic criteria.
